# Supplementary material for: Identification of Freezing-Responsive microRNAs and Their Targets in Chinese Jujube by Small RNA and Degradome Sequencing
Source: Int J Mol Sci. 2026 Sep 4;27(17):7912. doi: 10.3390/ijms27177912 (PMC13566890; doi:10.3390/ijms27177912)
Supplement: Supplementary file 1 [file ijms-27-07912-s001.zip › Supplementary Material S2/novel_miR_218.pdf]

Provisional ID	:	novel_miR_218
Score total	:	0.3
Score for star read(s)	:	-1.3
Score for read counts	:	0
Score for mfe	:	0
Score for randfold	:	1.6
Score for cons. seed	:	
Total read count	:	1732
Mature read count	:	1655
Loop read count	:	1
Star read count	:	76

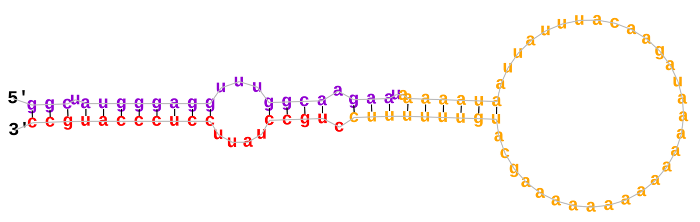

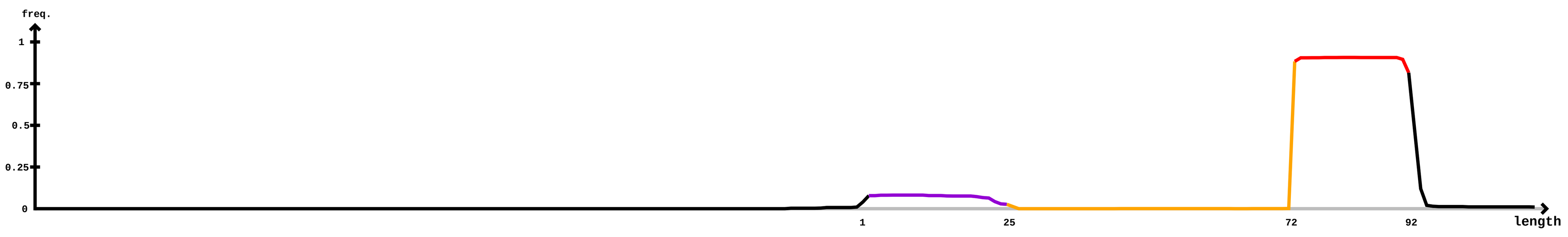

Star   Mature

```
5'- …gaacuuauggaaauuuuuggcuaugggagguuuggcaagaauaaaaauaauuauuuacaagauaaaaaaaaaaaaaaagcauguuuuuuccugccuauuccucccaugccaaugauuuccuuuuguuccucu -3'  obs
    …gaacuuauggaaauuuuuggcuaugggagguuuggcaagaauaaaaauaauuauuuacaagauaaaaaaaaaaaaaaagcauguuuuuuccugccuauuccucccaugccaaugauuuccuuuuguuccucu       exp
    …((((..(.(((((((.(((((.((((((((...((((.(((..((((((............................)))))))))).))))....))))))))))))).))))))).)..))))....
```

read	reads	mm	sample
uauggaaauuuuuggcuauggga	3	0	JCK-2
uauggaaauuuuuggcuauggga	2	0	JCK-3
aaauuuuuggcuaugggaggu	1	0	LT-3
aaauuuuuggcuaugggagguu	1	0	LT-3
aauuuuuggcuaugggaggu	3	0	LT-1
aauuuuuggcuaugggagguuuggc	3	0	LT-2
uuggcuaugggagguuuggc	4	0	LT-2
Cuggcuaugggagguuuggcaag	1	1	JCK-3
uggcuaugggagguuuggca	2	0	JCK-1
uggcuaugggagguuuggca	2	0	LT-1
uggcuaugggagguuuggca	1	0	JCK-3
uggcuaugggagguuuggca	1	0	LCK-3
uggcuaugggagguuuggca	1	0	JCK-2
uggcuaugggagguuuggcaa	3	0	LT-3
uggcuaugggagguuuggcaa	2	0	JCK-3
uggcuaugggagguuuggcaag	1	0	JCK-1
uggcuaugggagguuuggcaag	2	0	JCK-2
uggcuaugggagguuuggcaag	13	0	JT-1
uggcuaugggagguuuggcaag	2	0	LT-3
uggcuaugggagguuuggcaag	5	0	JCK-3
uggcuaugggagguuuggcaaga	4	0	JCK-2
uggcuaugggagguuuggcaaga	2	0	LT-3
uggcuaugggagguuuggcaaga	3	0	JCK-3
uggcuaugggagguuuggcaaga	6	0	JT-1
uggcuaugggagguuuggcaaga	3	0	JCK-1
uggcuaugggagguuuggcaagaUu	2	1	JCK-1
ggcuaugggagguuuggca	3	0	LCK-3
ggcuaugggagguuuggcaag	1	0	JCK-3
ggcuaugggagguuuggcaag	14	0	JT-1
ggcuaugggagguuuggcaaga	1	0	JCK-2
ggcuaugggagguuuggcaaga	1	0	JCK-3
ggcuaugggagguuuggcaaga	3	0	JCK-1
ggcuaugggagguuuggcaaga	2	0	LT-3

```
                                                                                                                                                 Star                                                                   Mature
nnnnnnnnnnnnnnnnnnnnnnnnnnnnnnnnnnnnnnnnnnnnnnnnnnnnnnnnnnnnnnnnnnnnnnnnnnnnnnnnnnnnnnnnnnnnnnnnnnnnnnnnnnnnnnnnnnnnnnnngaacuuauggaaauuuuuggcuaugggagguuuggcaagaauaaaaauaauuauuuacaagauaaaaaaaaaaaaaagcauguuuuuuccugccuauuccucccaugccaaugauuuccuuuuguuccucu
..........................................................................................................................................ggcuaugggagguuuggcaagaUu.........................................................................................   25   1   LCK-1
..........................................................................................................................................ggcuaugggagguuuggcaagaUu.........................................................................................   17   1   LCK-2
..........................................................................................................................................ggcuaugggagguuuggcaagaauU........................................................................................   2   1   JCK-3
..........................................................................................................................................ggcuaugggagguuuggcaagaauU........................................................................................   1   1   JCK-2
............................................................................................................................................cuaugggagguuuggcaagaa..........................................................................................   2   0   JCK-3
............................................................................................................................................cuaugggagguuuggcaagaa..........................................................................................   3   0   JCK-2
..............................................................................................................................................augggagguuuggcaagaUu.........................................................................................   1   1   JCK-3
....................................................................................................................................................................................Cauaaaaaaaaaaaaaagc....................................................   1   1   LT-3
..........................................................................................................................................................................................................uuuuuuccugccuauucc...............................   1   0   LT-3
................................................................................................................................................................................................................ccugccuauuccucccaugccaaug..................   1   0   LT-3
.................................................................................................................................................................................................................cugccuauuccucccauU........................   1   1   LT-3
.................................................................................................................................................................................................................cugccuauuccucccaug........................   2   0   JCK-3
.................................................................................................................................................................................................................cugccuauuccucccaug........................   13   0   LCK-3
.................................................................................................................................................................................................................cugccuauuccucccaug........................   3   0   JCK-2
.................................................................................................................................................................................................................cugccuauuccucccaug........................   1   0   LT-3
.................................................................................................................................................................................................................cugccuauuccucccaugA.......................   1   1   LT-1
.................................................................................................................................................................................................................cugccuauuccucccaugc.......................   2   0   JCK-3
.................................................................................................................................................................................................................cugccuauuccucccaugc.......................   24   0   LT-3
.................................................................................................................................................................................................................cugccuauucUucccaugc.......................   12   1   LCK-3
.................................................................................................................................................................................................................cugccuauuccucccaugc.......................   40   0   LCK-3
.................................................................................................................................................................................................................cugccuauuccucccaugc.......................   6   0   JCK-1
.................................................................................................................................................................................................................cugccuauuccucccaugc.......................   25   0   LT-1
.................................................................................................................................................................................................................cugccuCuuccucccaugc.......................   1   1   LT-3
.................................................................................................................................................................................................................cugccuauuccucccaugU.......................   4   1   LCK-3
.................................................................................................................................................................................................................cugccuauuccucccaugc.......................   8   0   LT-2
.................................................................................................................................................................................................................Uugccuauuccucccaugc.......................   1   1   LT-1
.................................................................................................................................................................................................................cugccuauuccucccaugc.......................   7   0   JT-1
.................................................................................................................................................................................................................cugccuauuccucccaugc.......................   6   0   JCK-2
.................................................................................................................................................................................................................cugccuCuuccucccaugc.......................   1   1   LCK-3
.................................................................................................................................................................................................................cugccuauuccucccaugcc......................   121   0   LT-3
.................................................................................................................................................................................................................cugccuauuccucccaugcc......................   160   0   LCK-3
.................................................................................................................................................................................................................cugccuauuccucccaugcc......................   31   0   JCK-1
.................................................................................................................................................................................................................cugccuauuccucccaugcA......................   2   1   LT-2
.................................................................................................................................................................................................................cugccuauuccucccaugcU......................   5   1   LCK-3
.................................................................................................................................................................................................................Uugccuauuccucccaugcc......................   1   1   LT-3
.................................................................................................................................................................................................................cugccuauuccucccaugcU......................   2   1   LT-2
.................................................................................................................................................................................................................cugccuauuccucccaugcU......................   6   1   LT-1
.................................................................................................................................................................................................................cugccuauuccucccaugcc......................   53   0   JT-1
.................................................................................................................................................................................................................cugccuCuuccucccaugcc......................   1   1   LCK-3
.................................................................................................................................................................................................................cugccuauuccucccaugcU......................   1   1   JCK-3
.................................................................................................................................................................................................................cugccuauuccucccaugcc......................   100   0   LT-1
.................................................................................................................................................................................................................cugccuauuccuUccaugcc......................   2   1   JCK-3
.................................................................................................................................................................................................................cugccuauuccucccaugcc......................   8   0   JT-3
.................................................................................................................................................................................................................cugccuauuccucccaugcU......................   8   1   JCK-2
.................................................................................................................................................................................................................cugccuauuccucccaugcA......................   2   1   LT-3
.................................................................................................................................................................................................................cuNccuauuccucccaugcc......................   1   1   LT-2
.................................................................................................................................................................................................................cugccuauuccucccaugUc......................   1   1   LT-1
.................................................................................................................................................................................................................cugccuauuccucccaugcU......................   6   1   LT-3
.................................................................................................................................................................................................................cugccuauuccucccaugcc......................   2   0   JT-2
.................................................................................................................................................................................................................cugccuauuccucccaugcc......................   46   0   JCK-2
.................................................................................................................................................................................................................cugccuauuccucccaugcc......................   73   0   LT-2
.................................................................................................................................................................................................................cugccuauuccucccaugcc......................   31   0   JCK-3
.................................................................................................................................................................................................................cugccuauuccucccaugcU......................   2   1   JCK-1
.................................................................................................................................................................................................................cugccuauuccucccaugcca.....................   81   0   LT-3
.................................................................................................................................................................................................................cugccuauuccucccaugccC.....................   1   1   LCK-3
.................................................................................................................................................................................................................cugccuauuccucccaugcca.....................   61   0   JCK-3
.................................................................................................................................................................................................................cugccuCuuccucccaugcca.....................   1   1   JCK-1
.................................................................................................................................................................................................................cugccuauuccucccaugcca.....................   11   0   JT-1
.................................................................................................................................................................................................................cugccuauuccucccaugcca.....................   33   0   LT-2
.................................................................................................................................................................................................................cugccuauuccucccaugcca.....................   66   0   LT-1
.................................................................................................................................................................................................................cugccuCuuccucccaugcca.....................   1   1   LCK-3
.................................................................................................................................................................................................................cugccuauuccucccaugccU.....................   1   1   LT-1
.................................................................................................................................................................................................................cugccuauuccNcccaugcca.....................   1   1   JCK-1
.................................................................................................................................................................................................................Uugccuauuccucccaugcca.....................   1   1   JCK-3
.................................................................................................................................................................................................................cugccuauuccucccaugcca.....................   57   0   LCK-3
.................................................................................................................................................................................................................cugccuauuccucccaugcca.....................   44   0   LCK-1
.................................................................................................................................................................................................................cugccuauuccucccaugcca.....................   1   0   JT-2
.................................................................................................................................................................................................................cugccuauuccucccaugccC.....................   1   1   LT-1
.................................................................................................................................................................................................................cugccuauuccucccaugcca.....................   68   0   LCK-2
.................................................................................................................................................................................................................Uugccuauuccucccaugcca.....................   2   1   JCK-1
```

```
                                                                                                                                             Star                                                                            Mature
nnnnnnnnnnnnnnnnnnnnnnnnnnnnnnnnnnnnnnnnnnnnnnnnnnnnnnnnnnnnnnnnnnnnnnnnnnnnnnnnnnnnnnnnnnnnnnnnnnnnnnnnnnnnnnnnnnnnnnngaacuuauggaaauuuuuggcuaugggagguuuggcaagaauaaaaauaauuauuuacaagauaaaaaaaaaaaaaaagcauguuuuuuccugccuauuccucccaugccaaugauuuccuuuuguuccucu
.................................................................................................................................................................................................................cugccuauuccucccaugcca.....................    9    0    JT-3
.................................................................................................................................................................................................................cugccuauuccucccaugcca.....................    46   0    JCK-1
.................................................................................................................................................................................................................cugccuauuccucNcaugcca.....................    1    1    LCK-1
.................................................................................................................................................................................................................cugccuauuccucccaugccU.....................    1    1    LT-3
.................................................................................................................................................................................................................cugccuCuuccucccaugcca.....................    1    1    JCK-2
.................................................................................................................................................................................................................cugccuauuccucccaugcca.....................    94   0    JCK-2
.................................................................................................................................................................................................................cugccuauuccucccaugccCa....................    1    1    JCK-1
.................................................................................................................................................................................................................cugccuauuccucccaugccaa....................    2    0    LT-2
.................................................................................................................................................................................................................cugccuauuccucccaugccaa....................    53   0    JCK-3
.................................................................................................................................................................................................................cugccuauuccucccaugccaU....................    1    1    LT-1
.................................................................................................................................................................................................................cugccuauuccucccaugccaa....................    8    0    JT-1
.................................................................................................................................................................................................................cugccuauuccucccaugccaa....................    19   0    LCK-3
.................................................................................................................................................................................................................cugccuauuccucccaugccaC....................    1    1    JCK-2
.................................................................................................................................................................................................................cugccuauuUcucccaugccaa....................    1    1    JCK-3
.................................................................................................................................................................................................................cugccuauuccucccaugccaa....................    12   0    LT-1
.................................................................................................................................................................................................................cugccuauuccucccaugccaa....................    27   0    JCK-1
.................................................................................................................................................................................................................cugccuauuccucccaugccaa....................    4    0    LT-3
.................................................................................................................................................................................................................cNgccuauuccucccaugccaa....................    1    1    JCK-2
.................................................................................................................................................................................................................cugccuauuccucccaugccaa....................    66   0    JCK-2
.................................................................................................................................................................................................................cugccuauuccucccaugccaUu...................    1    1    JCK-1
.................................................................................................................................................................................................................cugccuauuccucccaugccaau...................    1    0    JCK-2
.................................................................................................................................................................................................................cugccuauuccucccaugccaUu...................    2    1    JCK-3
.................................................................................................................................................................................................................cugccuauuccucccaugccaUu...................    2    1    LT-1
.................................................................................................................................................................................................................cugccuauuUcucccaugccaau...................    2    1    JCK-1
.................................................................................................................................................................................................................cugccuauuccucccaugccaaC...................    1    1    LT-1
.................................................................................................................................................................................................................cugccuauuccucccaugccaauU..................    1    1    LT-3
.................................................................................................................................................................................................................cugccuauuccucccaugccaauU..................    1    1    JCK-3
.................................................................................................................................................................................................................cugccuauuccucccaugccaauU..................    1    1    JCK-2
..................................................................................................................................................................................................................ugccuauuccucccaugc.......................    1    0    LT-2
..................................................................................................................................................................................................................ugccuauuccucccaugc.......................    7    0    LCK-3
..................................................................................................................................................................................................................ugccuauuccucccaugcc......................    2    0    LT-2
..................................................................................................................................................................................................................ugccuauuccucccaugcc......................    1    0    LT-1
..................................................................................................................................................................................................................ugccuauuccucccaugccU.....................    1    1    LT-1
..................................................................................................................................................................................................................ugccuauuccucccaugcca.....................    9    0    LCK-3
..................................................................................................................................................................................................................ugccuauuccucccaugcca.....................    3    0    JCK-2
..................................................................................................................................................................................................................ugccuauuccucccaugcca.....................    2    0    LT-3
..................................................................................................................................................................................................................ugccuauuccucccaugcca.....................    6    0    LT-1
..................................................................................................................................................................................................................ugccuauuccucccaugccaa....................    4    0    JCK-2
..................................................................................................................................................................................................................ugccuauuccucccaugccaa....................    1    0    JCK-3
..................................................................................................................................................................................................................ugccuauuccucccaugccaa....................    1    0    JCK-1
..................................................................................................................................................................................................................ugccuauuccucccaugccaau...................    1    0    LT-1
....................................................................................................................................................................................................................ccuauuccucccaugcca.....................    1    0    LT-3
......................................................................................................................................................................................................................uauuccucccaugccaaugauuuc.............    1    0    JCK-3
......................................................................................................................................................................................................................uauuccucccaugccaaugauuuc.............    1    0    JCK-1
.........................................................................................................................................................................................................................uccucccaugccaaugauuuc.............    1    0    JCK-3
.......................................................................................................................................................................................................................................ugauuuccuuuuguuccu..    1    0    JCK-2
.......................................................................................................................................................................................................................................ugauuuccuuuuguuccu..    1    0    LT-3
.......................................................................................................................................................................................................................................ugauuuccuuuuguuccuc.    4    0    LT-3
.......................................................................................................................................................................................................................................ugauuuccuuuuguuccuc.    3    0    JCK-1
.......................................................................................................................................................................................................................................ugauuuccuuuuguuccuc.    5    0    LT-2
.......................................................................................................................................................................................................................................ugauuuccuuuuguuccuc.    1    0    LT-1
.......................................................................................................................................................................................................................................ugauuuccuuuuguuccuc.    1    0    JCK-2
.......................................................................................................................................................................................................................................ugauuuccuuuuguuccucu    1    0    LCK-3
.......................................................................................................................................................................................................................................ugauuuccuuuuguuccucu    1    0    JCK-3
.......................................................................................................................................................................................................................................ugauuuccuuuuguuccucA    1    1    LT-2
.......................................................................................................................................................................................................................................ugauuuccuuuuguuccucu    1    0    JCK-1
```
